# PLOD1 Drives Head and Neck Squamous Cell Carcinoma Progression Through P4HA2‐Mediated Activation of the FAK/PI3K/AKT/mTOR Axis

**DOI:** 10.1111/jcmm.71275

**Published:** 2026-07-03

**Authors:** Yan‐Ling Wu, Hui Bai, Chao Jiang, Yuping Zhang, Wan Liu, JunJie Hang, Ying Huang

**Affiliations:** ^1^ Department of Radiation Oncology, National Cancer Center/National Clinical Research Center for Cancer/Cancer Hospital and Shenzhen Hospital Chinese Academy of Medical Sciences and Peking Union Medical College Shenzhen Guangdong China; ^2^ Department of Radiology Sichuan Clinical Research Center for Cancer, Sichuan Cancer Hospital & Institute, Sichuan Cancer Center, University of Electronic Science and Technology of China Chengdu China; ^3^ Department of Radiation Oncology, The People's Hospital of Shenzhen Baoan District The Second Affiliated Hospital of Shenzhen University Shenzhen China; ^4^ Department of Oncology The Second Affiliated Hospital of Shenzhen University (People's Hospital of Shenzhen Baoan District) Shenzhen Guang Dong China; ^5^ Department of Head and Neck Surgery, National Cancer Center/National Clinical Research Center for Cancer/Cancer Hospital & Shenzhen Hospital Chinese Academy of Medical Sciences and Peking Union Medical College Shenzhen Guangdong China; ^6^ Department of Oncology, National Cancer Center/National Clinical Research Center for Cancer/Cancer Hospital & Shenzhen Hospital Chinese Academy of Medical Sciences and Peking Union Medical College Shenzhen Guangdong China; ^7^ Department of Radiation Oncology State Key Laboratory of Oncology in South China, Guangdong Key Laboratory of Nasopharyngeal Carcinoma Diagnosis and Therapy, Guangdong Provincial Clinical Research Center for Cancer, Sun Yat‐sen University Cancer Center Guangzhou China

**Keywords:** FAK/PI3K/AKT/mTOR signalling, head and neck squamous cell carcinoma, P4HA2, PLOD1, prognostic biomarker

## Abstract

Some subtypes of head and neck squamous cell carcinoma (HNSCC) exhibit aggressive progression and poor prognosis, underscoring the need for novel therapeutic targets. While procollagen‐lysine, 2‐oxoglutarate 5‐dioxygenase 1 (PLOD1) is implicated in tumour collagen remodelling, its functional role and regulatory mechanisms in HNSCC remain elusive. PLOD1 expression and clinical relevance were analysed using TCGA‐HNSC data, patient tissues and cell lines. Functional impacts were assessed via in vitro assays (CCK‐8, flow cytometry, Transwell) and in vivo xenograft models. Mechanistic insights were explored through co‐immunoprecipitation, Western blotting, bioinformatics and pharmacological inhibition. PLOD1 was significantly upregulated in HNSCC tissues and correlated with adverse clinical outcomes. In vitro, PLOD1 overexpression potentiated proliferation, invasion and cell cycle progression while suppressing apoptosis; PLOD1 knockdown elicited opposing effects. PLOD1 activated the FAK/PI3K/AKT/mTOR pathway and directly interacted with prolyl 4‐hydroxylase subunit alpha 2 (P4HA2). P4HA2 rescue reversed PLOD1 knockdown‐mediated suppression of oncogenicity and pathway activation. The FAK inhibitor Y15 abrogated PLOD1‐driven malignant phenotypes. In vivo, PLOD1 silencing inhibited tumour growth and reduced FAK/PI3K/AKT/mTOR phosphorylation. PLOD1 drives HNSCC progression by modulating P4HA2 and activating the FAK/PI3K/AKT/mTOR signalling cascade, positioning the PLOD1‐P4HA2 axis as a promising prognostic biomarker and therapeutic target.

## 1 Introduction

Head and neck tumours encompass a diverse group of malignancies that can originate in regions such as the neck, ear, nasal cavity, throat and oral‐maxillofacial area. The majority of these cancers develop from the mucosal epithelial lining of the oral cavity, pharynx and larynx. Notably, over 90% of head and neck cancers are classified as squamous cell carcinomas, which originate from the epidermal or skin appendage tissues [1, 2, 3]. Epidemiological data from the Global Cancer Observatory indicate that the incidence of Head and Neck Squamous Cell Carcinoma (HNSCC) has been on the rise, with approximately 1.08 million new cases anticipated annually [4]. In 2021, HNSCC was projected to contribute to 78% of all head and neck cancer‐related deaths, and both incidence and mortality rates show a continued upward trend [1]. Most patients are diagnosed at an advanced local stage, resulting in a generally unfavourable prognosis. Approximately 50%–60% of patients experience local recurrence, and 20%–30% develop distant metastases within 2 years. The overall five‐year survival rate remains below 50% [5]. Consequently, there is a pressing need to discover novel and reliable molecular biomarkers to facilitate early detection, improve prognostic assessment and develop more effective therapeutic strategies for HNSCC.

Collagen, the most abundant structural protein in the ECM, exhibits extensive cross‐linking and aggregation, which markedly increases the rigidity of tumour tissues. This enhanced stiffness facilitates tumour cell proliferation, motility, invasion and metastatic potential [6, 7]. The post‐translational modification and stabilization of collagen fibres are heavily reliant on the activity of procollagen‐lysine, 2‐oxoglutarate 5‐dioxygenases (PLODs), which mediate collagen cross‐linking and ECM maturation [8]. Among the PLOD family, PLOD1‐located on chromosome 1p36.2‐36.3 and comprising 19 exons‐catalyses the hydroxylation of lysine residues in collagen molecules [9]. The resulting hydroxylysine sites serve as critical anchors for glycosylation, thereby enhancing the stability and robustness of intermolecular cross‐links. Consequently, PLOD1 is recognized for its role in promoting collagen fibre deposition and the stabilization of cross‐linking [10]. Recent studies have increasingly implicated PLODs in the pathogenesis and progression of various cancers, including endocrine‐related malignancies [11, 12, 13]. Notably, elevated expression of PLOD family genes has been correlated with unfavourable outcomes in gastric cancer patients [14]. Wang et al. [15] further demonstrated the involvement of PLOD1 in cancer progression. Despite these findings, the precise function and regulatory mechanisms of PLOD1 in HNSCC remain insufficiently characterized.

This study identifies dysregulated PLOD1 high expression in HNSCC, which correlates significantly with adverse clinical outcomes. Mechanistically, PLOD1 interacts with P4HA2 to potentiate HNSCC oncogenicity through FAK/PI3K/AKT/mTOR signalling cascade activation. These insights provide a foundation for developing PLOD1‐targeted therapeutic strategies.

## 2 Materials and Methods

### 2.1 Bioinformatic Analysis

Transcriptomic profiles and clinical metadata for HNSCC were retrieved from The Cancer Genome Atlas (TCGA) portal (gdc.cancer.gov). Expression values were log2‐transformed for downstream analyses. For survival and correlation analyses, patients were stratified into high/low expression groups using the median expression value of each gene as the unbiased cutoff and outlier expression values outside were filtered out prior to statistical analysis. Diagnostic accuracy of PLOD1 was determined through receiver operating characteristic (ROC) curve analysis implemented with the pROC package in R. Survival outcomes were modelled using Cox proportional hazards regression (survival package), with proportional hazards assumptions verified and results visualized via survminer/ggplot2. Spearman's rank correlation assessed gene expression relationships. PLOD1‐centred protein–protein interaction networks were constructed using STRING (string‐db.org). Functional enrichment analyses—incorporating Gene Ontology (GO), KEGG pathways and Gene Set Enrichment Analysis (GSEA)—were executed with clusterProfiler. All computational workflows utilized R version 4.2.1.

### 2.2 Specimen Collection

Thirty‐six tissue samples from patients with HNSCC were obtained at the Shenzhen Hospital, National Cancer Center/Cancer Hospital, Chinese Academy of Medical Sciences and Peking Union Medical College, between May 2021 and June 2023. When feasible, matched adjacent non‐tumorous tissue was collected as the normal control. Ethical approval for this study was granted by the Institutional Ethics Committee, with written consent provided by all participants. Surgical specimens underwent immediate cryopreservation in liquid nitrogen followed by storage at −80°C pending analysis.

### 2.3 Cell Culture and Reagents

Human HNSCC cell lines (CAL‐27, FaDu, SCC‐25; ATCC, USA), HEK293T cells (ATCC, USA) and normal oral keratinocytes (HOK; Tongpai, China) were cultured in DMEM (Gibco, USA) supplemented with 10% FBS (Gibco, USA) and 1% penicillin–streptomycin (Beyotime, China). All cell lines were incubated at 37°C under 5% CO_2_ humidified conditions. The focal adhesion kinase inhibitor Y15 was obtained from MedChemExpress (MCE, China).

### 2.4 Lentiviral Infection

Lentiviral constructs for PLOD1/P4HA2 overexpression and PLOD1 knockdown (shRNA) with corresponding controls (Vector/shCtrl) were sourced from GeneChem. Cells were seeded at 1 × 10^5^ cells/well (20%–30% confluence) and transduced using HiTransG P enhancer at MOI 10. Following 24‐h incubation, stable pools were selected in puromycin‐containing medium (2 μg/mL in 10% FBS/DMEM) for 7 days. Transduction efficiency was validated by RNA/protein analysis. The shPLOD1 target sequence was: 5′‐GCCGACTATTGACATCCACAT‐3′.

### 2.5 Western Blot Analysis

Total cellular protein was quantified with a BCA protein assay kit (Beyotime, China) after extraction using RIPA lysis buffer (Beyotime, China). Protein samples were resolved on 10% SDS‐polyacrylamide gels (Beyotime, China), denatured, and transferred to PVDF membranes (Bio‐Rad, USA). Membranes were blocked with 5% skim milk followed by overnight incubation at 4°C with primary antibodies: PLOD1 (1:1000, PA5‐61892, Invitrogen), P4HA2 (1:1000, ab211527, Abcam), FAK (1:2000, ab40794, Abcam), p‐FAK (1:2000, ab81298, Abcam), PI3K (1:1000, #4257, Cell Signalling), p‐PI3K (1:1000, #4257, Cell Signalling), AKT (1:500, AF6261, Affinity Biosciences), p‐AKT (1:500, AF0016, Affinity Biosciences) and GAPDH (1:1000, ab8245, Abcam). After 1‐h incubation with secondary antibodies, bands were detected using an ECL system (Bio‐Rad, USA), with exposure time strictly controlled between 10 s and 5 min to maintain linear signal range and avoid saturation. GAPDH expression served as loading control. Band densities were analysed with ImageJ software, with rolling ball background subtraction applied to eliminate non‐specific background noise and relative protein levels were normalized to GAPDH.

### 2.6 Real‐Time Quantitative PCR (qRT‐PCR)

RNA extraction was conducted with TRIzol reagent (Takara, Japan), and cDNA was subsequently generated using the PrimeScript RT kit. Quantitative PCR amplifications employed SYBR Green Master Mix (Takara) per manufacturer specifications. Cycle threshold (Ct) values were determined with standardized settings: baseline correction applied to cycles 3–15, fluorescence threshold set to 0.2 in the exponential amplification phase. Relative transcript levels were determined via the 2^−ΔΔCt^ method using GAPDH for normalization, with the average ΔCt of all matched adjacent normal tissues used as the calibrator. Samples with target gene Ct > 35 or technical replicate Ct standard deviation > 0.3 were excluded and re‐analysed for quality control. Corresponding primer sequences appear in Table 1.

**TABLE 1:** The primers utilized for RT‐PCR analysis of mRNA levels.

Target ID	Primer sequence, 5′‐3′
PLOD1	F: AGGCGGAACACACCTTTATG
	R: GAGAGCACGAGTCACTACAAAG
P4HA2	F: CAAACTGGTGAAGCGGCTAAA
	R: GCACAGAGAGGTTGGCGATA
GAPDH	F: CACCCACTCCTCCACCTTTG
	R: CCACCACCCTGTTGCTGTAG

### 2.7 Cell Proliferation Assay

Cell proliferation was assessed using the Cell Counting Kit‐8 (CCK‐8). Cells were trypsinized and seeded into 96‐well plates at a density of 1000 cells per well. Following incubation periods of 0, 24, 48, 72 and 96 h, 10 μL of CCK‐8 reagent was added to each well, and plates were incubated for 1 h at 37°C. Absorbance was then measured at 450 nm using a microplate reader.

### 2.8 Apoptosis and Cell Cycle Analysis

Apoptotic rates and cell cycle profiling were evaluated via flow cytometry following standardized protocols. Cells seeded at 1 × 10^6^ cells/well in 6‐well plates were harvested upon reaching confluence. After dual PBS rinses, apoptosis was measured using an Annexin V‐FITC/PI kit (YEASEN, China) on a Beckman Coulter CytoFLEX platform. Cell cycle synchronization involved 24‐h serum deprivation prior to 48‐h culture in complete medium. Fixed overnight in 70% ethanol at −20°C, samples were subsequently stained with PI/RNase buffer (20 min, RT). FlowJo (v10.6.2) processed all cytometry data.

### 2.9 Transwell Invasion Assay

Transwell invasion assays employed Matrigel‐coated chambers (24‐well format; Corning, USA). Serum‐starved cell suspensions (3 × 10^4^ cells/well) were loaded into upper compartments, with lower chambers containing 10% FBS medium to establish chemoattractive gradients. Following 24 h incubation, membrane‐invading cells underwent fixation, 0.5% crystal violet staining and light microscopic enumeration.

### 2.10 Animal Studies

Six‐week‐old male BALB/c nude mice were purchased from Vital River (Beijing, China) and housed in a specific pathogen‐free (SPF) environment. All animal procedures were performed in accordance with the guidelines approved by the Experimental Animal Institutional Review Board of Sun Yat‐sen University Cancer Center. Mice were randomly divided into shNC and shPLOD1 groups (*n* = 3 per group). A total of 1 × 10^7^ SCC‐25 cells suspended in 100 μL PBS were subcutaneously injected into each mouse. Tumour volumes were measured weekly with a vernier calliper; the longest (L) and shortest (W) diameters were recorded and tumour volume was calculated as (L × W^2^)/2. After 4 weeks, mice were euthanized, and tumours were dissected and weighed. Tissue samples were snap‐frozen in liquid nitrogen for subsequent Western blot analysis.

### 2.11 Statistical Analysis

Results are expressed as means ± standard deviations (SD). Statistical computations were conducted in SPSS (v22.0; IBM), with all experiments independently replicated at least three times. Bioinformatics analyses employed R (v4.21). Intergroup comparisons utilized Student's *t*‐test (two groups) or one‐way ANOVA (≥ 3 groups), with statistical significance defined as *p* < 0.05.

## 3 Results

### 3.1 Expression, Prognostic and Diagnostic Significance of PLOD1 in HNSCC

**FIGURE 1:**
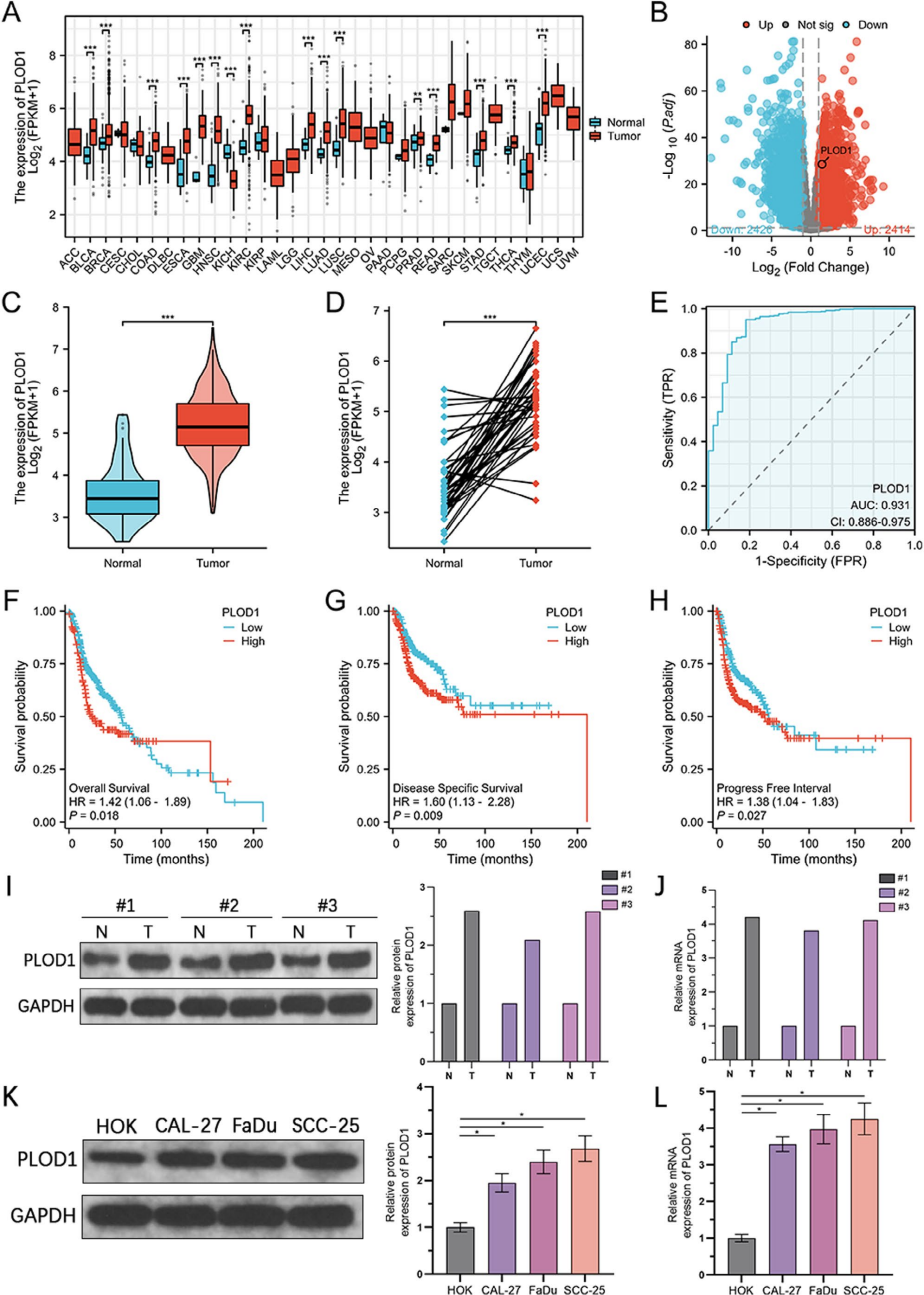
Expression, prognostic and diagnostic significance of PLOD1 in HNSCC. (A) Pan‐cancer analysis reveals differential PLOD1 expression across multiple tumour types. (B) Volcano plot depicting differentially expressed genes in the TCGA‐HNSC dataset. (C, D) Comparison of PLOD1 levels between HNSCC tumours and paired/unpaired adjacent non‐tumorous tissues from the TCGA‐HNSC cohort. (E) ROC curve analysis evaluating the diagnostic utility of PLOD1 for discriminating HNSCC from normal tissue. (F‐H) Kaplan–Meier curves illustrating OS, DSS and PFI stratified by PLOD1 expression in HNSCC patients. (I, J) WB and qRT‐PCR analysis of PLOD1 expression in HNSCC tissues versus adjacent tissues. (K, L) WB and qRT‐PCR assessment of PLOD1 levels in CAL‐27, FaDu, SCC‐25 (HNSCC) and HOK (normal oral keratinocyte) cell lines. *p < 0.05; **p < 0.01; ***p < 0.001.

Analysis of the TCGA pan‐cancer dataset revealed that PLOD1 mRNA expression was increased across most tumour types compared to their corresponding normal tissues (Figure 1A). Specifically in HNSCC, TCGA data indicated a marked upregulation of PLOD1 in cancerous tissues relative to adjacent non‐tumour samples (Figure 1B–D). ROC analysis evaluated PLOD1's diagnostic capacity in HNSCC, generating an AUC value of 0.931 (Figure 1E). Kaplan–Meier survival analysis demonstrated that elevated PLOD1 expression correlated with significantly diminished overall survival (OS: HR = 1.42, 95% CI: 1.06–1.89, 218 events; Figure 1F), compromised disease‐specific survival (DSS: HR = 1.60, 95% CI: 1.13–2.28, 130 events; Figure 1G) and shortened progression‐free interval (PFI: HR = 1.38, 95% CI: 1.04–1.83, 194 events; Figure 1H) in the TCGA‐HNSC cohort. Consistent with these findings, WB and qRT‐PCR analyses of HNSCC tissue samples confirmed elevated PLOD1 levels in tumours compared to matched normal tissues (Figure 1I,J). Furthermore, PLOD1 expression was examined in three HNSCC cell lines (CAL‐27, FaDu and SCC‐25) as well as in normal human oral keratinocytes (HOK), showing higher expression in all cancer cell lines, with the greatest elevation observed in SCC‐25 cells (Figure 1K,L).

### 3.2 Overexpression of PLOD1 Drives HNSCC Progression by Augmenting Proliferation, Cell Cycle Progression and Invasion While Inhibiting Apoptosis

**FIGURE 2:**
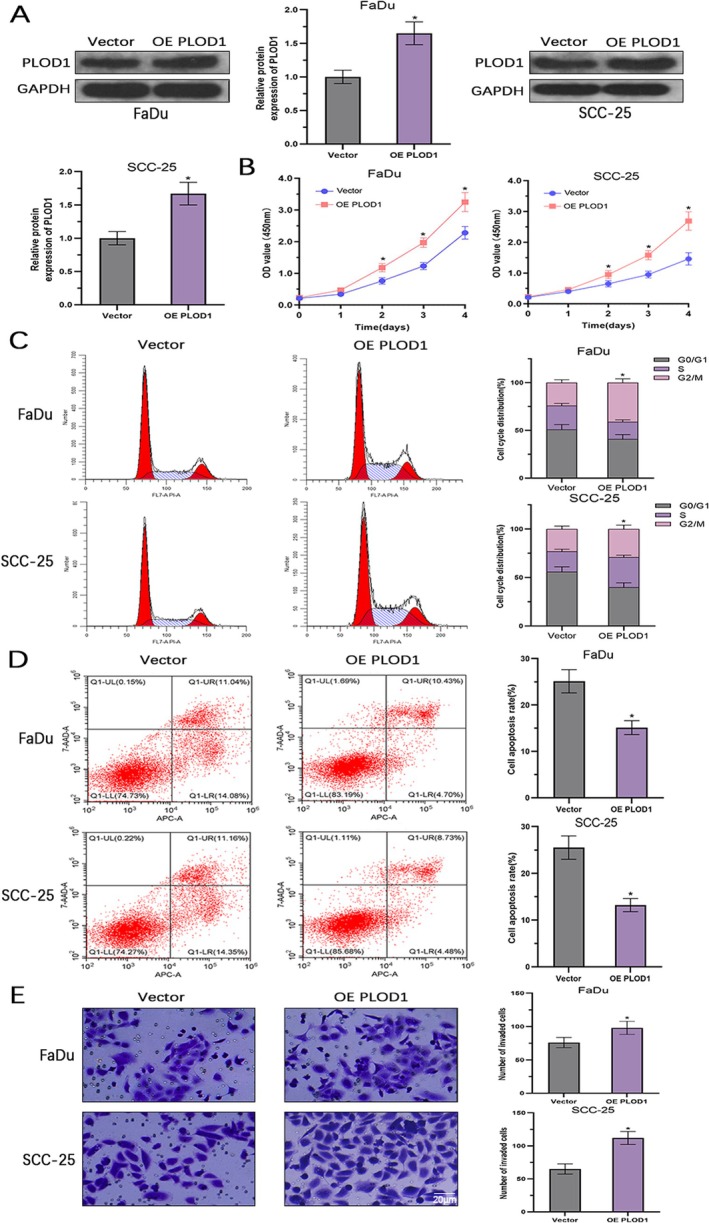
Overexpression of PLOD1 drives HNSCC progression by augmenting proliferation, cell cycle progression and invasion while inhibiting apoptosis. (A) WB confirming PLOD1 overexpression in FaDu and SCC‐25 cell lines. (B) Viability of FaDu and SCC‐25 cells assessed through CCK‐8 assay following PLOD1 overexpression. (C) Flow cytometric analysis of cell cycle distribution in FaDu and SCC‐25 cells with elevated PLOD1. (D) Quantification of apoptotic rates in FaDu and SCC‐25 cells via flow cytometry. (E) Transwell invasion assay evaluating the invasive capacity of FaDu and SCC‐25 cells upon PLOD1 upregulation. *p < 0.05.

To investigate the functional role of PLOD1 in driving HNSCC progression in vitro, we generated stable PLOD1‐overexpressing FaDu and SCC‐25 cell lines via lentiviral transduction. Successful overexpression was validated at the protein level (Figure 2A). Subsequent CCK‐8 assays demonstrated that PLOD1 potentiated proliferative capacity in both cell lines, with significantly higher viability in PLOD1‐overexpressing groups versus vector controls (Figure 2B). Cell cycle profiling by flow cytometry revealed that PLOD1 upregulation reduced the proportion of cells in G0/G1 phase while increasing accumulation in G2/M phase (Figure 2C). Concordantly, Annexin V/PI co‐staining showed attenuated apoptosis in PLOD1‐overexpressing cells (Figure 2D). Moreover, transwell invasion assays indicated that upregulation of PLOD1 markedly promoted the invasive potential of FaDu and SCC‐25 cells (Figure 2E).

### 3.3 Depletion of PLOD1 Impedes HNSCC Progression by Curtailing Proliferation, Cell Cycle Progression and Invasion, Concomitant With Enhanced Apoptosis

**FIGURE 3:**
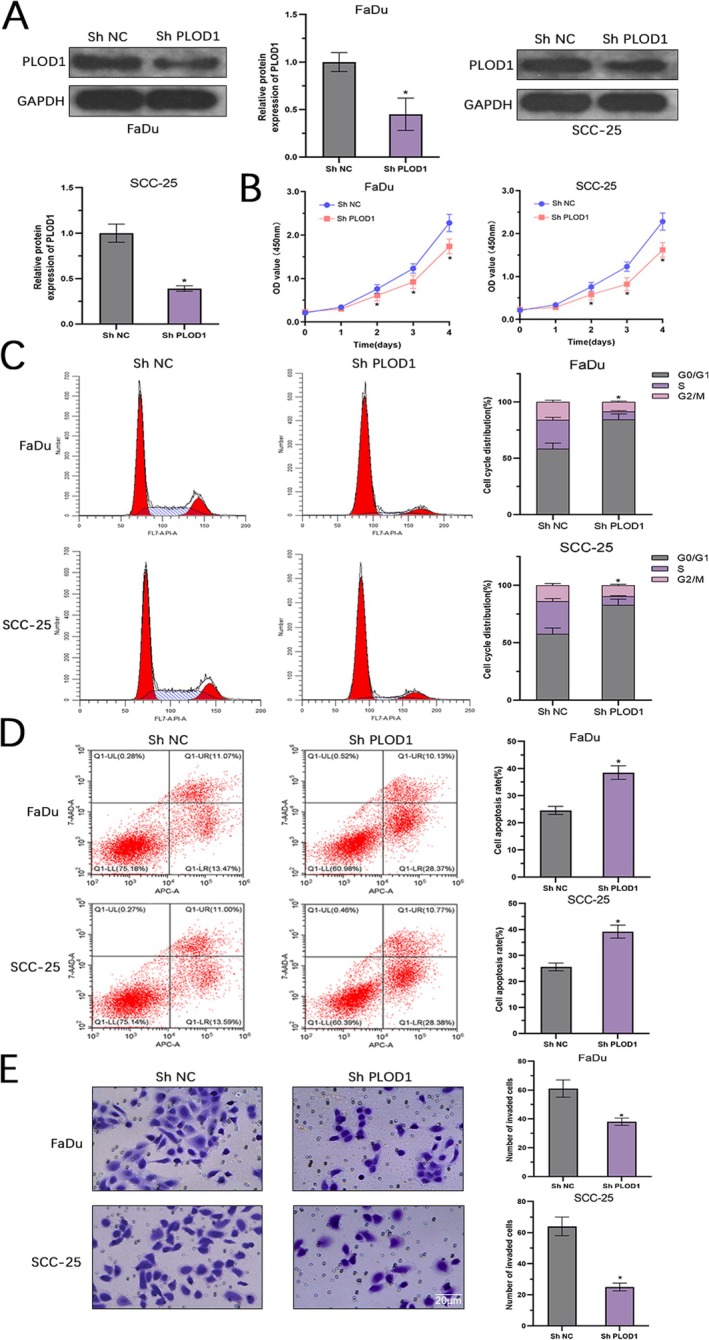
Depletion of PLOD1 impedes HNSCC progression by curtailing proliferation, cell cycle progression and invasion, concomitant with enhanced apoptosis. (A) Western blot validation of PLOD1 knockdown efficiency in FaDu and SCC‐25 cells. (B) Viability of FaDu and SCC‐25 cells assessed through CCK‐8 assay following PLOD1 silencing. (C) Flow cytometric evaluation of cell cycle phase distribution in FaDu and SCC‐25 cells upon PLOD1 silencing. (D) Flow cytometry‐based quantification of apoptotic rates in PLOD1‐silenced FaDu and SCC‐25 cells. (E) Transwell invasion assay measuring the invasive potential of FaDu and SCC‐25 cells after PLOD1 knockdown. *p < 0.05.

To delineate the tumour‐suppressive consequences of PLOD1 loss, we established stable PLOD1‐knockdown models in FaDu and SCC‐25 cells using shRNA lentivectors. Efficient silencing was confirmed by Western blotting (Figure 3A). Subsequent CCK‐8 assays revealed that PLOD1 ablation attenuated proliferative capacity in both cell lines, with significantly lower viability in PLOD1‐depleted groups versus scramble controls (Figure 3B). Cell cycle profiling by flow cytometry demonstrated that PLOD1 deficiency increased the proportion of cells in G0/G1 phase while reducing accumulation in G2/M phase (Figure 3C). Concordantly, Annexin V/PI co‐staining detected enhanced apoptosis in PLOD1‐depleted cells (Figure 3D). Moreover, transwell invasion assays indicated that suppression of PLOD1 substantially impaired the invasive potential of FaDu and SCC‐25 cells (Figure 3E).

### 3.4 Co‐Expression Network and Functional Enrichment of PLOD1‐Associated Genes in HNSCC

**FIGURE 4:**
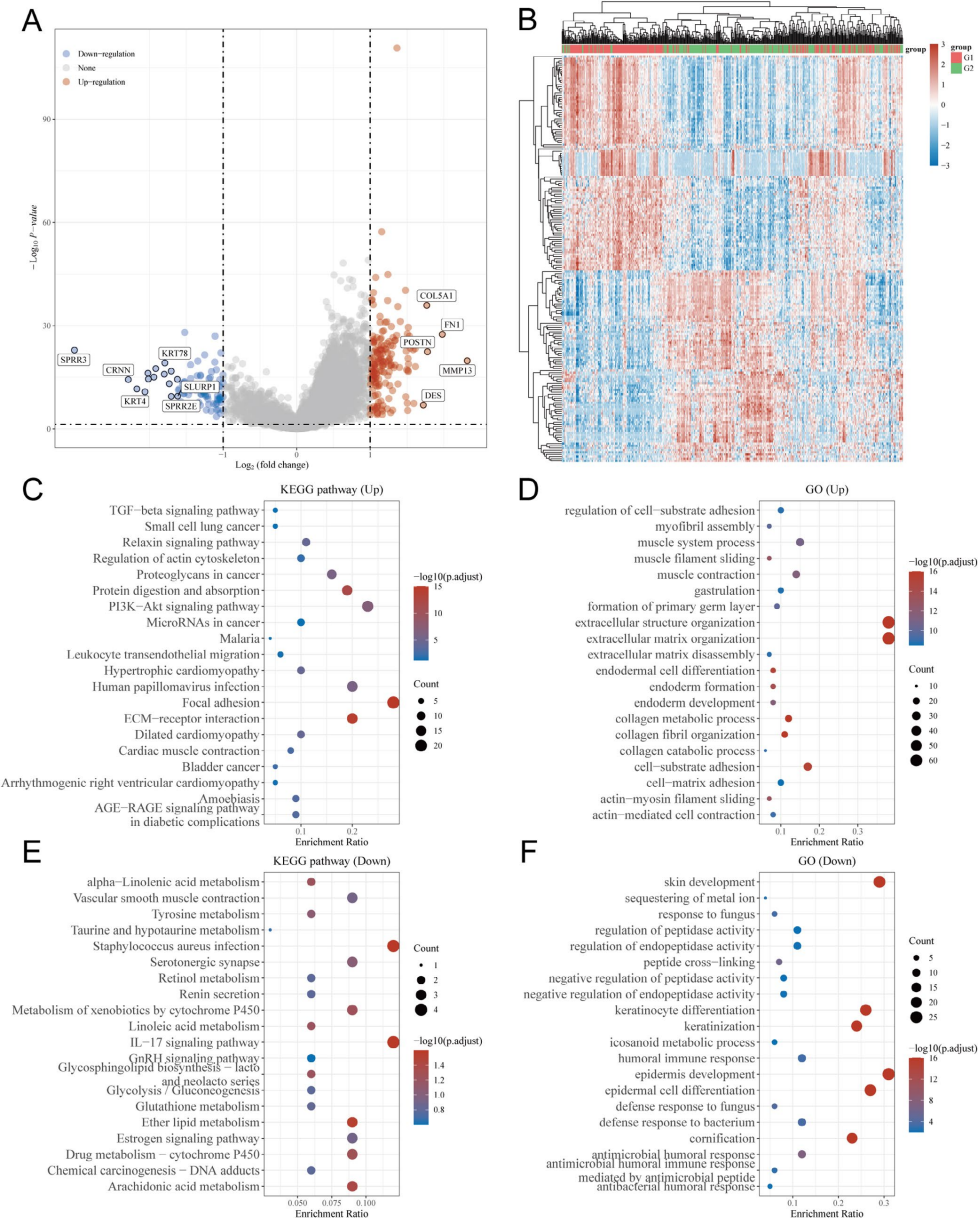
Co‐expression network and functional enrichment of PLOD1‐associated genes in HNSCC. (A, B) Volcano plot and heatmap analyses interrogating the TCGA‐HNSC dataset identified genes co‐expressed with PLOD1. (C, D) Genes positively correlating with PLOD1 underwent functional enrichment analysis, encompassing KEGG pathway and GO assessments. (E, F) KEGG and GO enrichment analyses were similarly applied to elucidate biological functions associated with genes inversely correlated with PLOD1. *p < 0.05.

To uncover the biological roles of PLOD1, transcriptomic data from the TCGA‐HNSCC dataset were analysed to identify genes that were significantly upregulated or downregulated and strongly correlated with PLOD1 expression levels (Figure 4A,B). These identified gene sets were then subjected to GO and KEGG pathway enrichment analyses. The results indicated that the upregulated genes were predominantly associated with pathways such as focal adhesion, PI3K/AKT signalling, extracellular matrix organization and extracellular structure organization (Figure 4C,D). In contrast, the enrichment analysis for the downregulated genes revealed significant involvement in processes including the IL‐17 signalling pathway, *Staphylococcus aureus* infection, epidermis development and skin development (Figure 4E,F).

### 3.5 PLOD1 Modulates HNSCC Progression via the FAK/PI3K/AKT/mTOR Pathway

**FIGURE 5:**
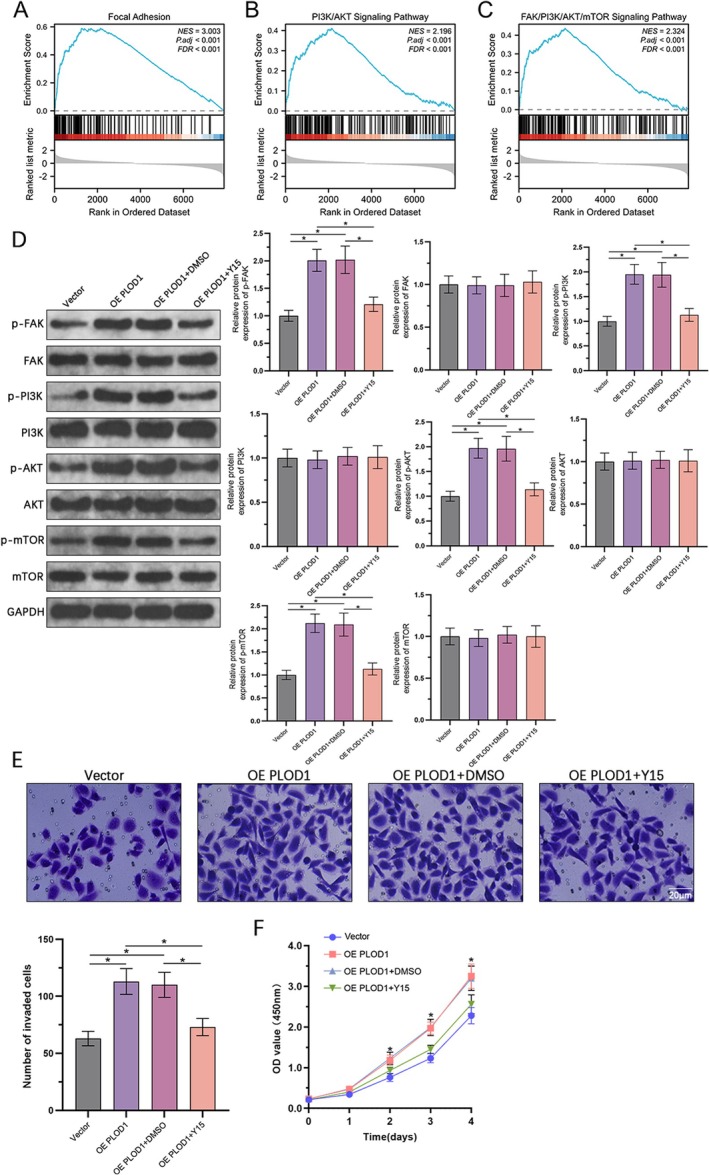
PLOD1 modulates HNSCC progression via the FAK/PI3K/AKT/mTOR pathway. (A–C) GSEA revealed a significant correlation between PLOD1 expression and the focal adhesion pathway, PI3K/AKT signalling, as well as the FAK/PI3K/AKT/mTOR cascade. (D) The expression levels of phosphorylated and total FAK, PI3K, AKT and mTOR proteins were evaluated in SCC‐25 cells after PLOD1 overexpression and subsequent Y15 treatment. (E, F) Invasion and proliferation of SCC‐25 cells were assessed via transwell and CCK‐8 assays following PLOD1 overexpression and subsequent Y15 treatment. *p < 0.05.

Analysis of TCGA‐HNSCC data using GSEA revealed that high PLOD1 expression in HNSCC samples was significantly associated with the enrichment of pathways such as focal adhesion, PI3K/AKT and FAK/PI3K/AKT/mTOR (Figure 5A–C). Furthermore, WB analysis in SCC‐25 cells demonstrated that PLOD1 upregulation led to increased phosphorylation levels of FAK, PI3K, AKT and mTOR. Treatment with the FAK inhibitor Y15 notably suppressed PLOD1‐induced phosphorylation of FAK, PI3K, AKT, and mTOR (Figure 5D). Additionally, Y15 was shown to significantly counteract the effects of PLOD1 overexpression on SCC‐25 cell invasion and proliferation (Figure 5E,F).

### 3.6 PLOD1 Modulates P4HA2 Expression and Interacts With It in HNSCC Cells

**FIGURE 6:**
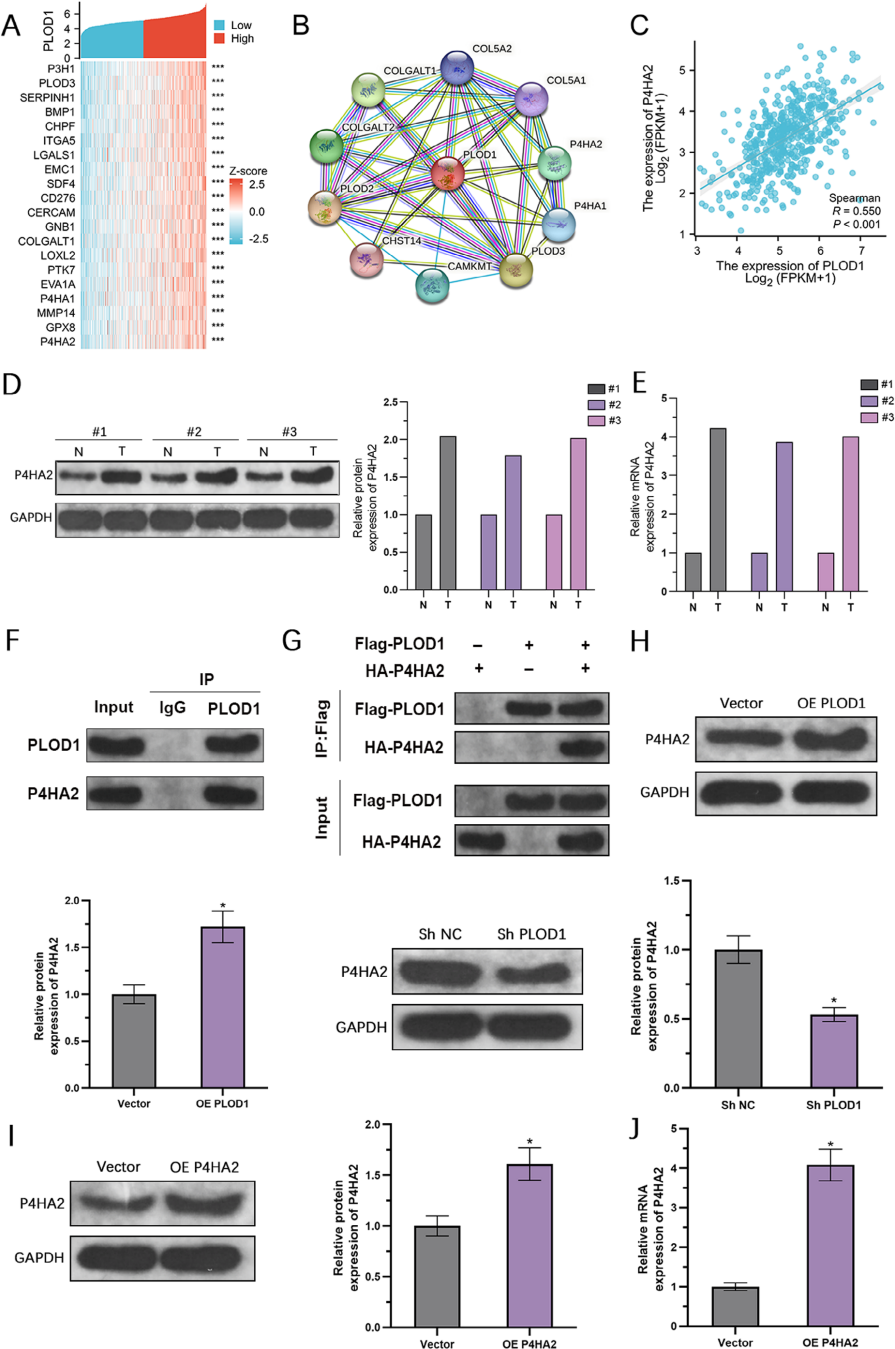
PLOD1 modulates P4HA2 expression and interacts with it in HNSCC cells. (A) The heatmap displays the top 20 genes showing the strongest positive correlation with PLOD1 in HNSCC samples. (B) PPI analysis using the STRING database demonstrated a link between PLOD1 and P4HA2. (C) Scatterplot analysis confirms significant co‐expression of PLOD1 and P4HA2 transcripts in HNSCC specimens. (D, E) WB and qRT‐PCR validate P4HA2 upregulation in HNSCC versus matched adjacent tissues. (F) Endogenous Co‐IP assays verified the interaction between PLOD1 and P4HA2 in SCC‐25 cells. (G) Exogenous Co‐IP experiments were performed in HEK293T cells transfected with the indicated constructs, further verifying the interaction between exogenous PLOD1 and P4HA2. (H) Western blot analysis was used to assess P4HA2 expression in SCC‐25 cells following either overexpression or knockdown of PLOD1. (I, J) The effects of PLOD1 overexpression were validated using both WB and qRT‐PCR assays. *p < 0.05.

To explore genes that are co‐expressed with PLOD1 in HNSCC, we performed an analysis using the TCGA‐HNSC dataset, with the goal of elucidating the molecular networks associated with PLOD1. Hierarchical clustering of the transcriptomic data was carried out, and the 20 genes most strongly correlated with PLOD1 expression were illustrated in a heatmap (Figure 6A). Subsequent STRING database analysis suggested a possible protein–protein interaction between PLOD1 and P4HA2 (Figure 6B). Cross‐validation analyses confirmed a significant positive correlation between the expression levels of PLOD1 and P4HA2 (Figure 6C). Both WB and qRT‐PCR demonstrated that P4HA2 was significantly upregulated in HNSCC tissues relative to adjacent non‐tumorous tissues (Figure 6D,E). Consequently, we further examined the direct association between PLOD1 and P4HA2. This interaction was verified through endogenous Co‐IP assays in SCC‐25 cells and exogenous Co‐IP in HEK293T cells (Figure 6F,G). In addition, WB analysis revealed that overexpressing PLOD1 in SCC‐25 cells led to a marked increase in P4HA2 levels, while silencing PLOD1 resulted in decreased P4HA2 expression (Figure 6H). Lentiviral‐mediated stable overexpression of PLOD1 in SCC‐25 cells was established, and successful modulation of PLOD1 was confirmed by both WB and qRT‐PCR (Figure 6I,J).

### 3.7 PLOD1 Promotes Invasive Behaviour, Cell Proliferation and Triggers Activation of the FAK/PI3K/AKT/mTOR Signalling Cascade Through Its Interaction With P4HA2 in HNSCC Cells

**FIGURE 7:**
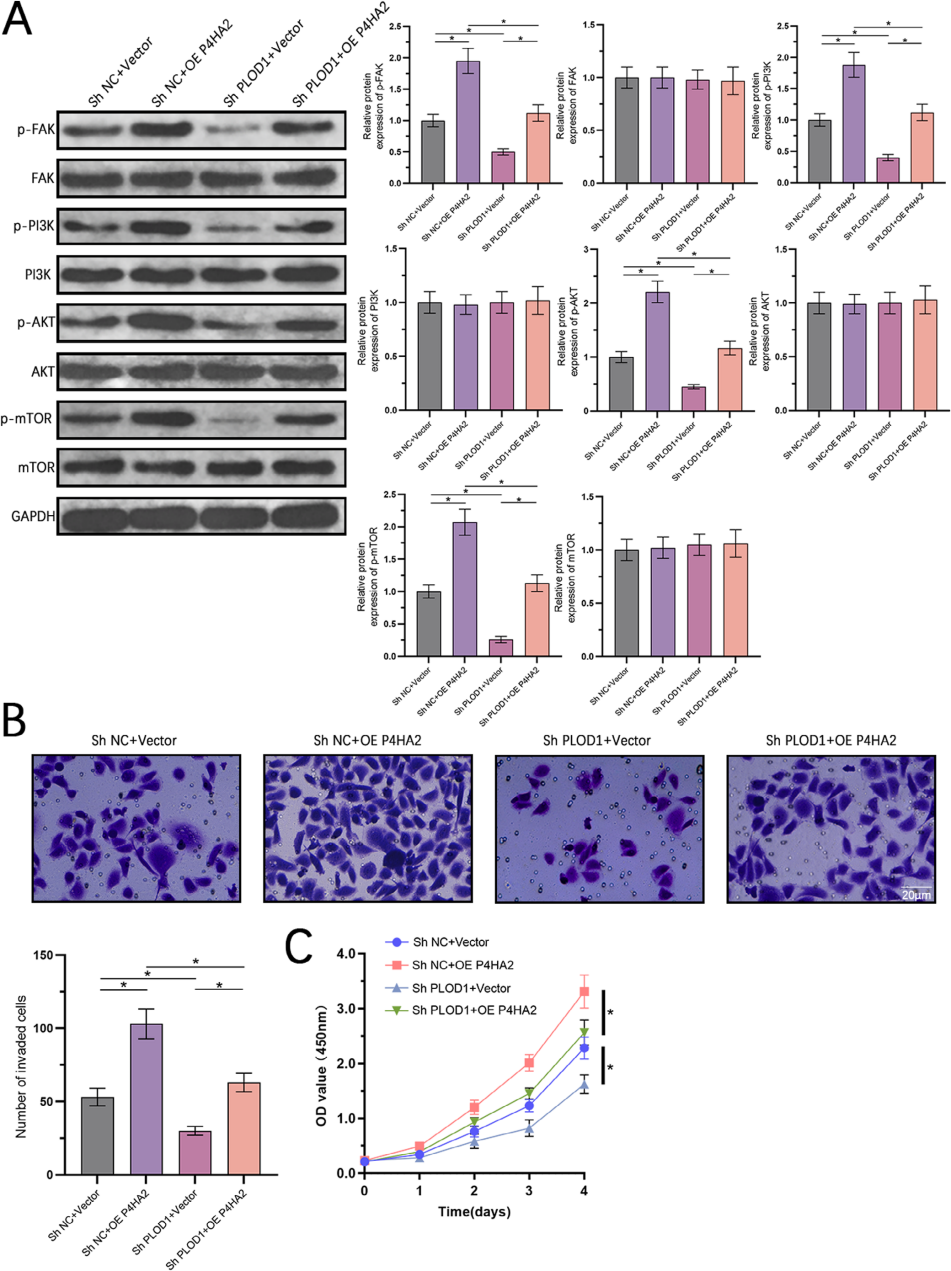
PLOD1 promotes invasive behaviour, cell proliferation and triggers activation of the FAK/PI3K/AKT/mTOR signalling cascade through its interaction with P4HA2 in HNSCC cells. (A) Western blot analysis was used to assess the expression levels of phosphorylated and total forms of FAK, PI3K, AKT and mTOR in SCC‐25 cells following PLOD1 knockdown and/or P4HA2 overexpression. (B) The invasive potential of SCC‐25 cells was analysed using the Transwell invasion assay. (C) Cell proliferation was quantified by the CCK‐8 assay. *p < 0.05.

Following validation of the PLOD1‐P4HA2 regulatory axis, we investigated its functional impact in HNSCC. WB analysis demonstrated that P4HA2 overexpression rescued PLOD1 knockdown‐induced suppression of FAK, PI3K, AKT and mTOR phosphorylation in SCC‐25 cells (Figure 7A). Transwell invasion assays further revealed that P4HA2 overexpression reversed the attenuated invasiveness resulting from PLOD1 silencing (Figure 7B). Concordantly, CCK‐8 assays showed P4HA2 overexpression significantly restored proliferation capacity impaired by PLOD1 depletion (Figure 7C).

### 3.8 PLOD1 Facilitates Tumour Development by Regulating P4HA2‐Mediated Activation of the FAK/PI3K/AKT/mTOR Pathway in HNSCC Xenograft Models

**FIGURE 8:**
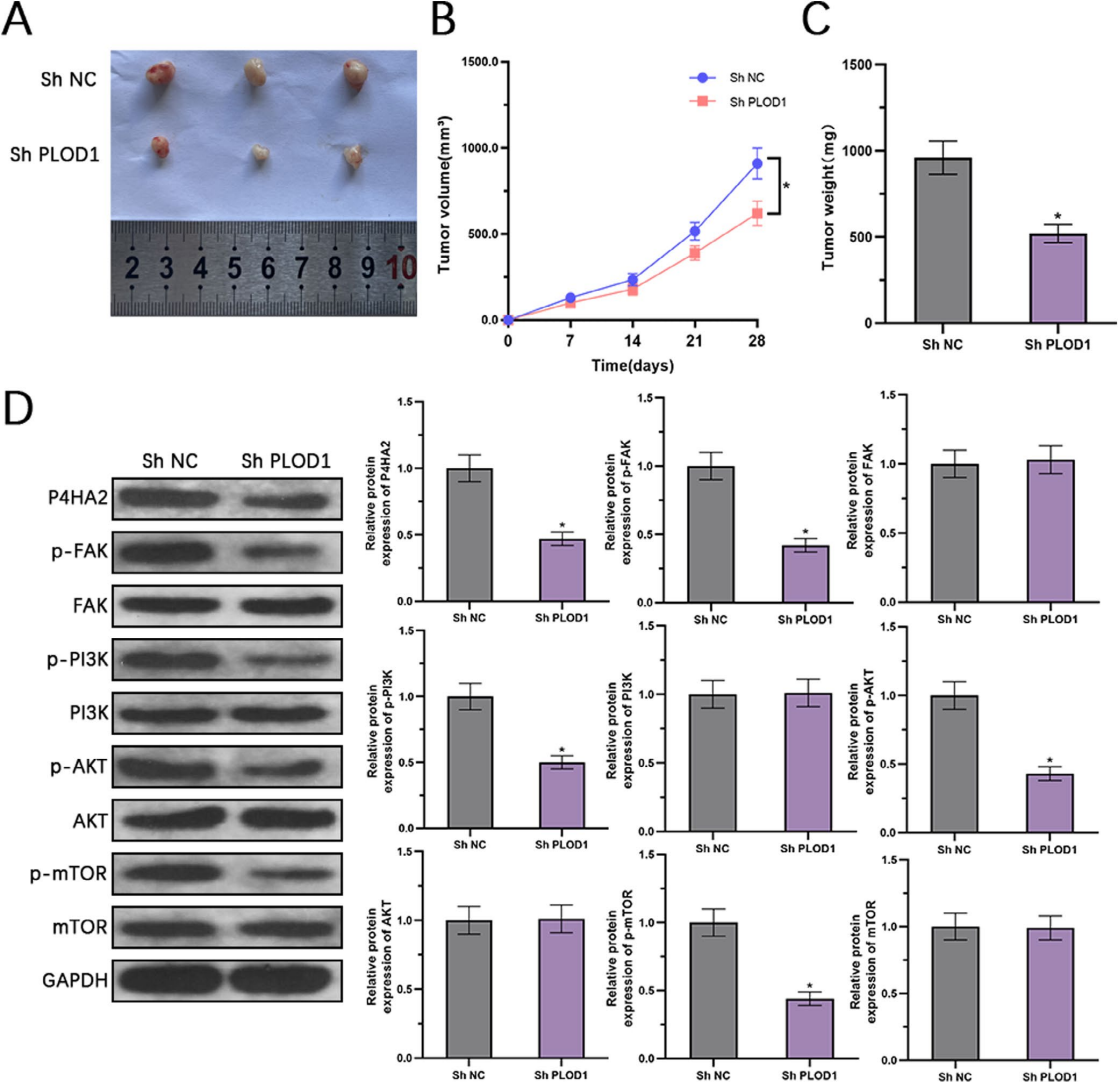
PLOD1 facilitates tumour development by regulating P4HA2‐mediated activation of the FAK/PI3K/AKT/mTOR pathway in HNSCC xenograft models. (A) Representative images show subcutaneous tumours formed in nude mice injected with either control SCC‐25 cells or SCC‐25 cells with stable PLOD1 knockdown. (B) Tumour volumes were measured and compared between the shNC (control) and shPLOD1 (PLOD1‐silenced) groups. (C) The weights of xenograft tumours from both groups were analysed. (D) Western blot analysis was performed to examine the expression levels of P4HA2 and phosphorylated and total forms of FAK, PI3K, AKT and mTOR in xenograft tumour tissues. *p < 0.05.

The impact of PLOD1 on HNSCC tumorigenesis was investigated in vivo utilizing xenograft models in mice. Knockdown of PLOD1 substantially inhibited tumour growth, as evidenced by significant reductions in both tumour volume and mass (Figure 8A–C). Subsequent WB analyses demonstrated that depletion of PLOD1 was associated with a notable decrease in the phosphorylation levels of FAK, PI3K, AKT and mTOR (Figure 8D).

## 4 Discussion

HNSCC, as a malignancy affecting multiple anatomical sites, is primarily marked by aggressive clinical features originating from mucosal epithelial transformation. These features include pronounced metastatic potential, invasiveness, immune cell infiltration and resistance to therapy [3, 16, 17]. Chronic infections caused by tumorigenic viruses such as HPV and EBV critically promote the pathogenesis of this disease. Additionally, exposure to risk factors like tobacco use and environmental pollutants significantly elevates the risk and poses a serious public health concern [16, 18]. The highly metastatic and immunosuppressive nature of HNSCC cells presents significant challenges for clinical intervention, highlighting the need to investigate the adaptive mechanisms underlying tumour cell survival in these contexts [3, 19]. Identifying novel molecular markers may improve both early detection and treatment efficacy in HNSCC [20]. Thus, advancing the search for reliable diagnostic and therapeutic biomarkers remains an urgent priority for enhancing patient outcomes.

Previous research has shown that PLOD1 plays a critical role in catalysing the hydroxylation of lysine residues, thereby facilitating collagen cross‐linking and accumulation. These processes are closely linked to tumorigenesis, as well as the regulation of cancer cell proliferation, apoptosis, invasion and migration [21, 22]. For instance, elevated PLOD1 levels in gastric cancer patients have been correlated with reduced OS and PFS^15^. Additionally, PLOD1 has been implicated in promoting aerobic glycolysis in gastric cancer cells [23]. In lung cancer, PLOD1 has been reported to drive tumorigenesis through activation of E2F1, suggesting a potential therapeutic target [24]. Furthermore, silencing PLOD1 expression has been shown to suppress the invasive capacity of bladder cancer cells and impede tumour progression [25]. Similarly, elevated PLOD1 levels correlate with enhanced malignancy progression in glioblastoma [26]. Bioinformatics analysis in this study identified upregulated PLOD1 expression in human HNSCC tissues compared to non‐tumour tissues. Elevated PLOD1 levels correlated with reduced survival duration and poorer clinical prognosis in HNSCC patients, indicating its potential role in tumour progression. In vitro functional assays demonstrated that PLOD1 overexpression promoted HNSCC cell proliferation, cell cycle progression and invasive capacity while inhibiting apoptosis. Conversely, PLOD1 knockdown exerted opposing effects. These observations align with prior reports of PLOD1's oncogenic functions in gastric cancer, bladder cancer and glioblastoma [23, 25, 26]. Collectively, PLOD1 represents an independent biomarker linked to aggressive clinical features in HNSCC.

Focal adhesion kinase (FAK), a non‐receptor tyrosine kinase, orchestrates cytoskeletal dynamics and is essential for cellular motility [27]. Signalling cascades downstream of FAK involve phosphatidylinositol‐3‐kinase (PI3K), protein kinase B (AKT) and mechanistic target of rapamycin (mTOR) [28, 29, 30, 31]. Hyperactivation of the PI3K‐AKT–mTOR axis through constitutive phosphorylation drives oncogenesis across multiple malignancies [32, 33]. Nevertheless, its mechanistic contribution to HNSCC pathogenesis remains incompletely elucidated. In this study, we demonstrate that forced expression of PLOD1 augments signalling along the FAK–PI3K–AKT–mTOR axis, whereas pharmacologic FAK blockade with Y15 attenuates this pathway in HNSCC cells. These findings indicate that PLOD1 functions upstream to potentiate FAK/PI3K/AKT/mTOR activation, thereby fostering aggressive phenotypes in HNSCC.

The gene prolyl 4‐hydroxylase subunit alpha 2 (P4HA2) encodes an α‐subunit of the prolyl 4‐hydroxylase holoenzyme that catalyses post‐translational proline hydroxylation in collagen [34, 35]. Owing to its pivotal position in collagen turnover, P4HA2 has been linked to diverse malignancies. In breast, glioma, ovarian and head and neck squamous cell carcinomas, elevated P4HA2 promotes metastasis through epithelial–mesenchymal transition (EMT) [36, 37, 38, 39]. Moreover, P4HA2 influences cancer development via the PI3K/AKT pathway and by activating endoplasmic reticulum stress programmes [39, 40, 41]. Seeking mechanistic insight into PLOD1‐mediated malignancy in HNSCC, we uncovered a cooperative interaction between PLOD1 and P4HA2. Overexpression of P4HA2 rescued the growth‐ and invasion‐inhibitory phenotypes caused by PLOD1 suppression and reinstated phosphorylation of FAK, PI3K, AKT and mTOR. Collectively, the data support a model in which the PLOD1–P4HA2 interaction promotes activation of the FAK/PI3K/AKT/mTOR signalling cascade during HNSCC progression.

Recent studies have proposed that cancer is essentially a multidimensional spatiotemporal pathological ecosystem of “unity of ecology and evolution”. Tumour metastasis and drug resistance are the outcomes of co‐evolution between tumour cells and their microenvironment, which cannot be explained by isolated molecular alterations alone [42, 43]. Based on our results, it is plausible that PLOD1‐mediated ECM remodelling may not only directly drive HNSCC malignant phenotypes, but also potentially contribute to constructing a pro‐tumorigenic ecological niche that exerts selection pressure for the evolution of more aggressive tumour clones. This eco‐evolutionary perspective provides a broader context for our findings, and further studies are warranted to validate this hypothesis.

## 5 Conclusion

PLOD1 drives HNSCC progression via P4HA2‐mediated FAK/PI3K/AKT/mTOR activation. Its overexpression correlates with poor prognosis and enhances malignant phenotypes. The PLOD1‐P4HA2 axis represents a prognostic biomarker and therapeutic target.

## Author Contributions

**Chao Jiang:** formal analysis, validation, visualization. **Yuping Zhang:** investigation, resources. **Hui Bai:** methodology, formal analysis, investigation. **JunJie Hang:** conceptualization, project administration, funding acquisition, writing – review and editing. **Yan‐Ling Wu:** investigation, data curation, writing – original draft. **Ying Huang:** conceptualization, supervision, methodology, writing – review and editing. **Wan Liu:** resources, data curation.

## Funding

The work was supported by grants from Sanming Project of Medicine in Shenzhen (SZSM202211030), Shenzhen Science and Technology Program (KCXFZ20211020172542002), and Shenzhen Medical Research Fund (C2401003).

## Ethics Statement

This research was approved by the Institutional Review Board (JS2024‐12‐1) of Cancer Hospital & Shenzhen Hospital, Chinese Academy of Medical Sciences and Peking Union Medical College, and the Experimental Animal Institutional Review Board (L102032024000F) of Sun Yat‐sen University Cancer Center.

## Consent

Prior written consent was well informed and signed by all participants.

## Conflicts of Interest

The authors declare no conflicts of interest.

## Data Availability

The data that support the findings of this study are available from the corresponding author upon reasonable request.
